# Supplementary material for: Human capital, gender, institutional environment and research funding: Determinants of research productivity in German psychology
Source: PLoS One. 2025 Feb 12;20(2):e0317673. doi: 10.1371/journal.pone.0317673 (PMC11819567; doi:10.1371/journal.pone.0317673)
Supplement: S1 Appendix — (DOCX) [file pone.0317673.s001.docx]

Appendix

# Summary statistics and t-test of accumulated variables for pre-doctorates, separately for females and males.

|  | Obs(m) | Obs(f) | Mean(m) | Mean(f) | dif | se | t-value |
| --- | --- | --- | --- | --- | --- | --- | --- |
| SSCI/SCIE articles | 335 | 606 | 1.02 | .74 | .28 | .07 | 3.95 |
| Monographs | 335 | 606 | 0.06 | .05 | 0 | .03 | .15 |
| Book chapters | 335 | 606 | 0.34 | .38 | -.04 | .06 | -.6 |
| Non-SSCIE/SCIE articles | 335 | 606 | 0.28 | .3 | -.02 | .04 | -.6 |
| Edited volumes | 335 | 606 | 0.00 | .01 | -.01 | 0 | -2.05 |
| Gray literature | 335 | 606 | 0.39 | .31 | .08 | .07 | 1.05 |
| Months abroad | 335 | 606 | 5.59 | 5.89 | -.29 | .9 | -.35 |
| Doctorate abroad | 335 | 606 | 0.02 | .01 | .01 | .01 | 1.7 |
| High-status university | 335 | 606 | 0.27 | .24 | .02 | .03 | .75 |
| Research funding | 335 | 606 | 0.01 | .01 | 0 | .01 | .1 |
| Mothers | 335 | 606 | 0.00 | .13 | -.13 | .02 | -7.1 |
| Fathers | 335 | 606 | 0.09 | 0 | .09 | .01 | 7.7 |
| Children status unknown (female) | 335 | 606 | 0.00 | .37 | -.37 | .03 | -14.05 |
| Children status unknown (male) | 335 | 606 | 1.02 | .74 | .28 | .07 | 3.95 |
|  |  |  |  |  |  |  |  |

# Summary statistics and t-test of accumulated variables for post-doctorates, separately for females and males.

|  | Obs(m) | Obs(f) | Mean(m) | Mean(f) | dif | se | t-value |
| --- | --- | --- | --- | --- | --- | --- | --- |
| SSCI/SCIE articles | 325 | 520 | 5.77 | 3.92 | 1.85 | .37 | 5 |
| Monographs | 325 | 520 | 0.30 | .23 | .07 | .04 | 1.55 |
| Book chapters | 325 | 520 | 1.96 | 1.59 | .37 | .22 | 1.7 |
| Non-SSCIE/SCIE articles | 325 | 520 | 1.23 | .99 | .24 | .17 | 1.45 |
| Edited volumes | 325 | 520 | 0.12 | .05 | .07 | .04 | 1.5 |
| Gray literature | 325 | 520 | 2.04 | .89 | 1.14 | .47 | 2.45 |
| Months abroad | 325 | 520 | 11.70 | 11.71 | -.01 | 1.69 | 0 |
| Doctorate abroad | 325 | 520 | 0.18 | .14 | .03 | .03 | 1.2 |
| High-status university | 325 | 520 | 0.21 | .25 | -.04 | .03 | -1.6 |
| Research funding | 325 | 520 | 0.33 | .19 | .14 | .04 | 3.3 |
| Mothers | 325 | 520 | 0.00 | .39 | -.39 | .03 | -14.3 |
| Fathers | 325 | 520 | 0.35 | 0 | .35 | .02 | 16.75 |
| Children status unknown (female) | 325 | 520 | 0.00 | .31 | -.31 | .03 | -12.15 |
| Children status unknown (male) | 325 | 520 | 0.41 | 0 | .41 | .02 | 19.1 |
|  |  |  |  |  |  |  |  |

# Summary statistics and t-test of accumulated variables for untenured assistant professors (habil/junior professor), separately for females and males.

|  | Obs(m) | Obs(f) | Mean(m) | Mean(f) | dif | se | t-value |
| --- | --- | --- | --- | --- | --- | --- | --- |
| SSCI/SCIE articles | 59 | 58 | 15.71 | 10.27 | 5.45 | 1.7 | 3.2 |
| Monographs | 59 | 58 | 0.72 | .9 | -.18 | .36 | -.5 |
| Book chapters | 59 | 58 | 6.10 | 4.91 | 1.19 | 1.65 | .7 |
| Non-SSCIE/SCIE articles | 59 | 58 | 3.02 | 2.22 | .8 | .7 | 1.15 |
| Edited volumes | 59 | 58 | 0.38 | .18 | .2 | .13 | 1.5 |
| Gray literature | 59 | 58 | 1.98 | 2 | -.01 | .67 | 0 |
| Months abroad | 59 | 58 | 13.66 | 12.36 | 1.3 | 4.32 | .3 |
| Doctorate abroad | 59 | 58 | 0.07 | .1 | -.04 | .05 | -.7 |
| High-status university | 59 | 58 | 0.27 | .31 | -.05 | .07 | -.65 |
| Research funding | 59 | 58 | 1.58 | .9 | .68 | .28 | 2.45 |
| Mothers | 59 | 58 | 0.00 | .45 | -.45 | .07 | -6.85 |
| Fathers | 59 | 58 | 0.39 | 0 | .39 | .06 | 6.05 |
| Children status unknown (female) | 59 | 58 | 0.00 | .38 | -.38 | .06 | -5.95 |
| Children status unknown (male) | 59 | 58 | 0.44 | 0 | .44 | .07 | 6.7 |

# Summary statistics and t-test of accumulated variables for tenured professors (W2/W3), separately for females and males.

|  | Obs(m) | Obs(f) | Mean(m) | Mean(f) | dif | se | t-value |
| --- | --- | --- | --- | --- | --- | --- | --- |
| SSCI/SCIE articles | 291 | 161 | 35.73 | 23.55 | 12.18 | 2.32 | 5.25 |
| Monographs | 291 | 161 | 1.63 | 1.46 | .17 | .26 | .65 |
| Book chapters | 291 | 161 | 16.77 | 13.18 | 3.6 | 1.91 | 1.9 |
| Non-SSCIE/SCIE articles | 291 | 161 | 7.52 | 5.73 | 1.79 | .84 | 2.1 |
| Edited volumes | 291 | 161 | 1.51 | .85 | .65 | .26 | 2.55 |
| Gray literature | 291 | 161 | 5.59 | 3.28 | 2.31 | .91 | 2.55 |
| Months abroad | 291 | 161 | 27.80 | 25.15 | 2.65 | 3.97 | .65 |
| Doctorate abroad | 291 | 161 | 0.10 | .11 | -.01 | .03 | -.4 |
| High-status university | 291 | 161 | 0.28 | .3 | -.02 | .03 | -.75 |
| Research funding | 291 | 161 | 4.02 | 2.7 | 1.32 | .42 | 3.15 |
| Mothers | 291 | 161 | 0.00 | .53 | -.53 | .03 | -18.25 |
| Fathers | 291 | 161 | 0.47 | 0 | .47 | .04 | 11.85 |
| Children status unknown (female) | 291 | 161 | 0.00 | .3 | -.3 | .03 | -11.25 |
| Children status unknown (male) | 291 | 161 | 0.43 | 0 | .43 | .04 | 10.9 |

# Random-effects models on annual SSCI/SCIE publications, different specifications

|  | (1) | (2) | (3) | (4) | (5) |
| --- | --- | --- | --- | --- | --- |
|  | full  model | articles  divided  author | articles  no  auth  adj | articles  x  imp  factor | complete  record |
| Women | -0.26^***^ | -0.31^***^ | -0.23^***^ | -0.26^***^ | -0.27^***^ |
|  | (-5.17) | (-5.15) | (-4.56) | (-5.17) | (-5.30) |
| Pre-docs | 0.00 | 0.00 | 0.00 | 0.00 | 0.00 |
|  | (.) | (.) | (.) | (.) | (.) |
| Post-docs | 0.40^***^ | 0.45^***^ | 0.39^***^ | 0.40^***^ | 0.44^***^ |
|  | (17.84) | (17.85) | (15.64) | (17.84) | (13.73) |
| Ass prof | 0.61^***^ | 0.68^***^ | 0.57^***^ | 0.61^***^ | 0.63^***^ |
|  | (10.86) | (11.24) | (8.91) | (10.86) | (8.65) |
| Tenured prof | 0.75^***^ | 0.79^***^ | 0.79^***^ | 0.75^***^ | 0.74^***^ |
|  | (9.90) | (9.27) | (9.68) | (9.90) | (6.66) |
| Months abroad (ln) | 0.11^***^ | 0.12^***^ | 0.11^***^ | 0.11^***^ | 0.11^***^ |
|  | (7.02) | (7.05) | (6.60) | (7.02) | (5.26) |
| Doctorate abroad | 0.03 | 0.02 | 0.04 | 0.03 | -0.00 |
|  | (0.46) | (0.27) | (0.60) | (0.46) | (-0.06) |
| High-status university | 0.10^**^ | 0.10^**^ | 0.14^**^ | 0.10^**^ | 0.12^*^ |
|  | (2.89) | (2.68) | (3.13) | (2.89) | (2.53) |
| Research funding (ln) | 0.67^***^ | 0.64^***^ | 0.88^***^ | 0.67^***^ | 0.66^***^ |
|  | (11.18) | (10.47) | (10.54) | (11.18) | (7.82) |
| Mothers | -0.22^***^ | -0.24^***^ | -0.22^***^ | -0.22^***^ | -0.22^***^ |
|  | (-4.70) | (-4.82) | (-4.08) | (-4.70) | (-4.44) |
| Fathers | -0.03 | -0.07 | 0.04 | -0.03 | -0.02 |
|  | (-0.45) | (-0.83) | (0.50) | (-0.45) | (-0.26) |
| Child=unknown (women) | -0.06 | -0.07^+^ | -0.04 | -0.06 | 0.00 |
|  | (-1.58) | (-1.71) | (-0.78) | (-1.58) | (.) |
| Child=unknown (men) | -0.13^*^ | -0.15^*^ | -0.11^+^ | -0.13^*^ | 0.00 |
|  | (-2.17) | (-2.16) | (-1.92) | (-2.17) | (.) |
| Selected publication list | -0.46^***^ | -0.49^***^ | -0.48^***^ | -0.46^***^ | -0.48^***^ |
|  | (-6.97) | (-7.03) | (-5.51) | (-6.97) | (-4.99) |
| Cohort<1990 | -0.08 | 0.00 | -0.25^***^ | -0.08 | -0.18^+^ |
|  | (-1.18) | (0.06) | (-3.34) | (-1.18) | (-1.77) |
| 1990-1999 | -0.06 | 0.00 | -0.20^***^ | -0.06 | -0.10^+^ |
|  | (-1.21) | (0.03) | (-3.67) | (-1.21) | (-1.72) |
| 2000-2009 | 0.09^**^ | 0.11^**^ | 0.07^+^ | 0.09^**^ | 0.12^**^ |
|  | (2.79) | (3.09) | (1.90) | (2.79) | (2.99) |
| Cohort>2009 | 0.00 | 0.00 | 0.00 | 0.00 | 0.00 |
|  | (.) | (.) | (.) | (.) | (.) |
| Constant | 1.06^***^ | 1.10^***^ | 1.18^***^ | 1.06^***^ | 1.06^***^ |
|  | (30.19) | (27.90) | (31.32) | (30.19) | (24.99) |
| R2 within | 0.16 | 0.13 | 0.20 | 0.16 | 0.15 |
| R2 between | 0.40 | 0.39 | 0.36 | 0.40 | 0.37 |
| R2 overall | 0.27 | 0.25 | 0.28 | 0.27 | 0.25 |
| Researcher | 2175 | 2175 | 2175 | 2175 | 1344 |
| Observations | 23300 | 23300 | 23300 | 23300 | 13693 |

t statistics in parentheses. Variables mean-centered, sd=1

+ p < 0.1, * p < 0.05, ** p < 0.01, *** p < 0.001

Table A6. Random-effects models on annual SSCI/SCIE publications, without controlling for prior publication activity, non-logged.

|  | (1) | (2) | (3) | (4) | (5) | (6) |
| --- | --- | --- | --- | --- | --- | --- |
|  | Baseline | Career | Funding | Parenting | Women only | Men only |
| Women | -0.34^***^ | -0.30^***^ | -0.28^***^ | -0.26^***^ |  |  |
|  | (-8.80) | (-9.04) | (-8.44) | (-5.00) |  |  |
| Pre-doc |  | 0.00 | 0.00 | 0.00 | 0.00 | 0.00 |
|  |  | (.) | (.) | (.) | (.) | (.) |
| Post-doc |  | 0.48^***^ | 0.44^***^ | 0.45^***^ | 0.37^***^ | 0.53^***^ |
|  |  | (21.58) | (20.22) | (20.39) | (15.17) | (14.91) |
| Ass prof |  | 0.87^***^ | 0.72^***^ | 0.75^***^ | 0.80^***^ | 0.75^***^ |
|  |  | (16.69) | (14.09) | (14.10) | (9.56) | (10.37) |
| Tenured prof |  | 1.32^***^ | 0.85^***^ | 0.88^***^ | 0.95^***^ | 0.89^***^ |
|  |  | (19.55) | (13.01) | (12.79) | (9.56) | (10.03) |
| Months abroad |  | 0.01^***^ | 0.01^***^ | 0.01^***^ | 0.01^***^ | 0.01^***^ |
|  |  | (5.74) | (6.20) | (6.18) | (5.02) | (4.62) |
| Doctorate abroad |  | -0.12^+^ | -0.09 | -0.08 | 0.04 | -0.15 |
|  |  | (-1.86) | (-1.38) | (-1.31) | (0.64) | (-1.49) |
| High-status uni |  | 0.12^**^ | 0.11^**^ | 0.11^**^ | 0.12^**^ | 0.11^+^ |
|  |  | (3.09) | (3.10) | (3.22) | (2.78) | (1.74) |
| Research funding |  |  | 0.18^***^ | 0.18^***^ | 0.16^***^ | 0.18^***^ |
|  |  |  | (10.68) | (10.72) | (5.72) | (9.24) |
| Mother |  |  |  | -0.18^***^ | -0.14^**^ | 0.00 |
|  |  |  |  | (-4.07) | (-2.99) | (.) |
| Father |  |  |  | 0.00 | 0.00 | -0.00 |
|  |  |  |  | (0.03) | (.) | (-0.04) |
| Child=unknown (women) |  |  |  | -0.07^+^ | -0.06 | 0.00 |
|  |  |  |  | (-1.88) | (-1.52) | (.) |
| Child=unknown (men) |  |  |  | -0.11^+^ | 0.00 | -0.12^*^ |
|  |  |  |  | (-1.83) | (.) | (-1.98) |
| Selected publications | -0.17^*^ | -0.46^***^ | -0.45^***^ | -0.46^***^ | -0.27^***^ | -0.58^***^ |
|  | (-2.28) | (-6.60) | (-6.86) | (-6.96) | (-3.34) | (-6.14) |
| Cohort<1990 | 0.56^***^ | -0.14^*^ | -0.17^**^ | -0.15^*^ | -0.37^***^ | -0.05 |
|  | (5.94) | (-1.99) | (-2.64) | (-2.31) | (-5.42) | (-0.64) |
| 1990-1999 | 0.54^***^ | -0.03 | -0.06 | -0.05 | -0.08 | -0.01 |
|  | (9.36) | (-0.56) | (-1.22) | (-0.97) | (-1.43) | (-0.16) |
| 2000-2009 | 0.39^***^ | 0.10^**^ | 0.09^**^ | 0.10^**^ | 0.06^+^ | 0.15^**^ |
|  | (10.01) | (2.93) | (2.96) | (3.21) | (1.87) | (2.63) |
| Cohort>2009 | 0.00 | 0.00 | 0.00 | 0.00 | 0.00 | 0.00 |
|  | (.) | (.) | (.) | (.) | (.) | (.) |
| Constant | 0.58^***^ | 0.99^***^ | 1.00^***^ | 1.05^***^ | 0.93^***^ | 1.13^***^ |
|  | (29.98) | (38.91) | (41.33) | (29.49) | (27.49) | (20.02) |
| R2 within | 0.00 | 0.13 | 0.17 | 0.17 | 0.12 | 0.19 |
| R2 between | 0.14 | 0.35 | 0.40 | 0.40 | 0.39 | 0.37 |
| R2 overall | 0.05 | 0.22 | 0.28 | 0.28 | 0.24 | 0.27 |
| Researcher | 2175 | 2175 | 2175 | 2175 | 1191 | 984 |
| Observations | 23300 | 23300 | 23300 | 23300 | 10528 | 12772 |

t statistics in parentheses. Variables mean-centered, sd=1

+ p < 0.1, * p < 0.05, ** p < 0.01, *** p < 0.001

Table A7. Random-effects models on annual SSCI/SCIE publications, without controlling for prior publication activity, non-logged.

|  | (1) | (2) | (3) | (4) | (5) | (6) |
| --- | --- | --- | --- | --- | --- | --- |
|  | Baseline | Career | Funding | Parenting | Women  only | Men  only |
| Women | -0.19^***^ | -0.20^***^ | -0.20^***^ | -0.26^***^ |  |  |
|  | (-7.32) | (-7.66) | (-7.65) | (-5.45) |  |  |
| SSCI/SCIE articles | 0.07^***^ | 0.06^***^ | 0.06^***^ | 0.06^***^ | 0.08^***^ | 0.06^***^ |
|  | (21.05) | (16.42) | (12.06) | (12.16) | (14.69) | (9.80) |
| Monographs | -0.02 | -0.03 | -0.03 | -0.03 | 0.01 | -0.04^+^ |
|  | (-1.18) | (-1.37) | (-1.40) | (-1.45) | (0.29) | (-1.69) |
| Book chapters | 0.01 | 0.00 | 0.00 | 0.00 | 0.00 | 0.00 |
|  | (1.33) | (0.84) | (0.78) | (0.88) | (0.62) | (0.74) |
| Non-SSCI/SCIE articles | -0.01 | -0.01^+^ | -0.01 | -0.01 | -0.02 | -0.00 |
|  | (-1.62) | (-1.80) | (-1.46) | (-1.36) | (-1.54) | (-0.63) |
| Edited volumes | -0.05^+^ | -0.03 | -0.03 | -0.04 | -0.01 | -0.04 |
|  | (-1.86) | (-1.29) | (-1.35) | (-1.55) | (-0.27) | (-1.31) |
| Gray literature | -0.01^*^ | -0.01^*^ | -0.01^*^ | -0.01^*^ | -0.02^**^ | -0.01^+^ |
|  | (-2.13) | (-2.19) | (-2.19) | (-2.02) | (-2.91) | (-1.78) |
| Pre-doc |  | 0.00 | 0.00 | 0.00 | 0.00 | 0.00 |
|  |  | (.) | (.) | (.) | (.) | (.) |
| Post-doc |  | 0.32^***^ | 0.32^***^ | 0.34^***^ | 0.25^***^ | 0.42^***^ |
|  |  | (15.67) | (15.76) | (16.90) | (10.57) | (12.76) |
| Ass prof |  | 0.48^***^ | 0.48^***^ | 0.51^***^ | 0.48^***^ | 0.50^***^ |
|  |  | (9.42) | (9.41) | (9.89) | (6.87) | (7.13) |
| Tenured prof |  | 0.40^***^ | 0.37^***^ | 0.41^***^ | 0.27^***^ | 0.41^***^ |
|  |  | (5.57) | (5.59) | (6.02) | (3.34) | (4.47) |
| Months abroad |  | 0.00^***^ | 0.00^***^ | 0.00^***^ | 0.00^*^ | 0.00^**^ |
|  |  | (3.79) | (3.94) | (3.87) | (2.47) | (2.99) |
| Doctorate abroad |  | -0.04 | -0.04 | -0.04 | 0.03 | -0.10 |
|  |  | (-0.84) | (-0.82) | (-0.90) | (0.84) | (-1.37) |
| High-status uni |  | 0.11^***^ | 0.11^***^ | 0.11^***^ | 0.09^**^ | 0.14^**^ |
|  |  | (3.34) | (3.38) | (3.54) | (2.68) | (2.75) |
| Research funding |  |  | 0.03 | 0.03 | 0.01 | 0.03 |
|  |  |  | (1.22) | (1.28) | (0.55) | (1.28) |
| Mother |  |  |  | -0.16^***^ | -0.16^***^ | 0.00 |
|  |  |  |  | (-3.85) | (-4.35) | (.) |
| Father |  |  |  | -0.17^*^ | 0.00 | -0.16^*^ |
|  |  |  |  | (-2.46) | (.) | (-2.19) |
| Child=unknown (women) |  |  |  | -0.05 | -0.06^*^ | 0.00 |
|  |  |  |  | (-1.41) | (-2.30) | (.) |
| Child=unknown (men) |  |  |  | -0.17^**^ | 0.00 | -0.16^**^ |
|  |  |  |  | (-3.13) | (.) | (-2.91) |
| Selected publications | -0.14^**^ | -0.22^***^ | -0.23^***^ | -0.22^***^ | -0.05 | -0.32^***^ |
|  | (-2.97) | (-4.33) | (-4.59) | (-4.58) | (-0.85) | (-4.78) |
| Cohort<1990 | -0.20^***^ | -0.34^***^ | -0.34^***^ | -0.32^***^ | -0.35^***^ | -0.27^***^ |
|  | (-4.87) | (-8.51) | (-8.44) | (-7.89) | (-8.51) | (-4.50) |
| 1990-1999 | 0.03 | -0.12^***^ | -0.12^***^ | -0.11^**^ | -0.16^***^ | -0.07 |
|  | (0.94) | (-3.46) | (-3.56) | (-3.25) | (-4.20) | (-1.34) |
| 2000-2009 | 0.18^***^ | 0.06^*^ | 0.06^*^ | 0.07^**^ | 0.02 | 0.12^*^ |
|  | (6.32) | (2.39) | (2.42) | (2.74) | (0.77) | (2.45) |
| Cohort>2009 | 0.00 | 0.00 | 0.00 | 0.00 | 0.00 | 0.00 |
|  | (.) | (.) | (.) | (.) | (.) | (.) |
| Constant | 0.96^***^ | 1.07^***^ | 1.07^***^ | 1.15^***^ | 1.09^***^ | 1.23^***^ |
|  | (43.48) | (51.06) | (51.09) | (34.27) | (38.96) | (22.10) |
| R2 within | 0.15 | 0.17 | 0.17 | 0.17 | 0.13 | 0.19 |
| R2 between | 0.71 | 0.70 | 0.70 | 0.70 | 0.70 | 0.71 |
| R2 overall | 0.38 | 0.40 | 0.40 | 0.40 | 0.36 | 0.40 |
| Researcher | 2175 | 2175 | 2175 | 2175 | 1191 | 984 |
| Observations | 23300 | 23300 | 23300 | 23300 | 10528 | 12772 |

t statistics in parentheses. Variables mean-centered, sd=1

+ p < 0.1, * p < 0.05, ** p < 0.01, *** p < 0.001
