# Supplementary material for: Human capital, gender, institutional environment and research funding: Determinants of research productivity in German psychology
Source: PLoS One. 2025 Feb 12;20(2):e0317673. doi: 10.1371/journal.pone.0317673 (PMC11819567; doi:10.1371/journal.pone.0317673)
Supplement: Attachment — Submitted filename: Response to Reviewers.docx [file pone.0317673.s002.docx]

# Response to Reviewers

# PONE-D-24-24540: “Human capital, gender, institutional environment and research funding: Determinants of research productivity in German psychology”

Dear editor and reviewers,

Thank you for of your thoughtful and useful suggestions, from which the paper has benefitted greatly. We have copied your suggestions below, detailing how we have taken up each.

**Editor comment**: Please clarify whether the study focuses on the sacred spark hypothesis, the cumulative advantage hypothesis, or integrates both. It would be helpful to clearly define the expected empirical patterns and hypotheses for each approach. Additionally, the role of the institutional environment, research funding, and gender should be more clearly addressed.

**Author response**: Reviewer 2 raised a similar point and we agree that the paper benefits from making this more explicit. We have clarified the framing accordingly, arguing that the paper tests which of the two human capital theories is correct, either the “sacred spark” hypothesis, which suggests that researchers are more productive irrespective of their prior productivity, or the “cumulative advantage” hypothesis, which suggests that researchers’ productivity is explicable through their prior experience in publishing articles. We now mention these two hypotheses explicitly and test them, showing and discussing that the cumulative advantage hypothesis explains research output better than the sacred spark hypothesis, as early experience strongly explains later productivity irrespective of time-invariant differences. This clearer framing also lets us conceptualize the other variables (gender, institutional environment and research funding) as confounders on the two hypotheses that are posited by the two human capital theories that we test.

**Editor comment**: Re-evaluate the selection of sources to ensure that they are relevant to the European context. Including more empirical findings from similar national contexts would strengthen the contextual relevance of your study.

**Author response**: Yes, we were lacking a bit in this part. We have now situated our research within the added studies of D'Amico et al., 2011; Helmreich et al., 1980; König et al., 2015 and for the context of German psychology we have added Habicht, 2023; Lutter et al., 2022; Mayer & Rathmann, 2018.

**Editor comment**: Consider the placement of international mobility within the institutional environment section. Discussing its wider implications, such as network expansion, may provide additional insights.

**Author response**: We have placed international mobility within the institutional environment section and mentioned that it may lead to network expansion.

**Editor comment**: Justify the choice of SSCI/SCIE articles and discuss the process of fractionalizing and weighting publications by journal impact factor in the main text.

**Author response**: We moved the discussion of fractionalizing and weighting publications by journal impact factor into the main text. We added three references to show how SSCI/SCIE articles are the main measure of productivity in psychology.

**Editor comment**: Please justify the inclusion of only university psychologists and discuss the potential impact of excluding researchers from other sectors (e.g., MPG institutes).

**Author response**: So far, we have only mentioned that we include “each of the two leading research institutes” that play an important role in German psychology. These are, exactly as you suggest, the two relevant MPG institutes, notably from the Max Planck Institute for Human Cognitive and Brain Sciences and the Max Planck Institute for Human Development. Yet for privacy protection reasons, we have been asked to not name these explicitly. We therefore now mention the “two relevant Max Planck Institutes in this field” as a compromise. We now also justify our strategy for including them and every German university with a psychology department, on the grounds that this comprises the virtually complete universe of German academic psychology, as universities of applied science are geared towards teaching rather than research.

**Editor comment**: A more detailed explanation of your methods and statistical analyses is needed. Including information on how you dealt with reciprocal causality and unknown confounders would be helpful.

**Author response**: Our FE models control for all time-invariant confounders, even unknown ones. To rule out time-varying confounders, we follow a theoretical framework on what confounders should be relevant. Yet, indeed we cannot account for time-varying confounders such as biological age (to the degree that it is unaccounted for by career stages), time-varying caring responsibilities (to the degree that it is not accounted for by gender and parental status) and personal characteristics such as career orientation. Not being able to account for all potentially relevant confounders is a problem that we share with all studies that regress on observables. We should have made this clearer, however. We therefore now mention what we cannot account for in the introduction as well when discussing the limitations of our study and make everything we have just written explicit (also see our response to reviewer below).

**Editor comment**: Ensure that all appendices (D, E, and F) are included in the document.

**Author response**: Indeed, some appendices were missing. This has been rectified.

**Editor comment**: Provide more detailed information on how mobility and staying abroad were measured, along with the reliability of these measurements.

**Author response**: We now mention that: “We measure international experience through a doctoral degree from an institution obtained outside Germany and through the number of months spent abroad, assuming five months for a semester and ten months for an academic year in case websites did not list exact months.” Please also see our response to the reviewer below. In case this is not sufficient, we would be glad to incorporate whatever extra information is required.

**Editor comment**: Consider distinguishing between different levels of professorship and career stages to account for heterogeneity within the professor category.

**Author response**: We have done this as suggested by the reviewer (see below).

**Editor comment**: Think about additional output indicators to capture the impact of research output.

**Author response**: We have multiplied all journal articles by the impact factor of the journal in which they appeared (cf. Appendix A5 Model 4 compared to Model 1). This yields essentially similar results.

Thank you for these thoughtful suggestions, from which the paper has profited enormously. As you could see, we think that we could incorporate all of them.

**Reviewer comment**: 1. Is the manuscript technically sound, and do the data support the conclusions?

The manuscript must describe a technically sound piece of scientific research with data that supports the conclusions. Experiments must have been conducted rigorously, with appropriate controls, replication, and sample sizes. The conclusions must be drawn appropriately based on the data presented.

Reviewer #1: Partly

Reviewer #2: Yes

2. Has the statistical analysis been performed appropriately and rigorously?

Reviewer #1: I Don't Know

Reviewer #2: Yes

3. Have the authors made all data underlying the findings in their manuscript fully available?

The [PLOS Data policy](http://www.plosone.org/static/policies.action#sharing) requires authors to make all data underlying the findings described in their manuscript fully available without restriction, with rare exception (please refer to the Data Availability Statement in the manuscript PDF file). The data should be provided as part of the manuscript or its supporting information, or deposited to a public repository. For example, in addition to summary statistics, the data points behind means, medians and variance measures should be available. If there are restrictions on publicly sharing data—e.g. participant privacy or use of data from a third party—those must be specified.

Reviewer #1: No

Reviewer #2: No

**Author response**:

The anonymized dataset and code to replicate the study analyses have been made available at OSF <https://osf.io/qdnb2/?view_only=dd06a0a9adf54f8e8d85992bc660bdda> For reasons of data protection, we have excluded variables on having children (additional survey).

**Reviewer comment**: 4. Is the manuscript presented in an intelligible fashion and written in standard English?

PLOS ONE does not copyedit accepted manuscripts, so the language in submitted articles must be clear, correct, and unambiguous. Any typographical or grammatical errors should be corrected at revision, so please note any specific errors here.

Reviewer #1: Yes

Reviewer #2: Yes

5. Review Comments to the Author

**Reviewer #1**: This is an interesting paper on an important issue, of interest to a large audience (although this is not a formal criterion).

The first problem is that the methods are described rather short, and that it is therefore not completely clear what was done and why. There is no code and no data to check things. A more informative explanation of the various steps in the analysis is required, with information what statistical software was used and with the code. Many things therefore remain somewhat unclear. For example, how was the reciprocal causality (performance <-> motherhood; earlier performance -> mobility (or grants) -> later performance) handled in the analysis. And the random and fixed effect analyses are used among other to control for unknown confounders - what variables not included could still have an effect on the findings, apart from of course disciplinary differences (as the study only includes psychology) and country differences.

**Author response**: These are very good points and taking them into account has significantly benefitted the paper. First, we have uploaded the updated code and data with the new submission. Second, we now mention that all analyses were done using Stata. Third, compared to existing studies that use cross-sectional data, we use longitudinal data with FE models, which allows to control for all time-invariant confounders. Our main way of dealing with reciprocal causality is that we know when something happened, e.g. we know at which point in time researchers became parents, published a new paper etc. This allows us to see whether a change at time t1 was followed by another change in time t2. We also use regressions that first *do not* and then *do* control for prior publications, thus measuring whether a researcher a) published more after certain conditions were in place and b) published more than would be expected given his or her publication trajectory until this point, which is akin to using a lagged panel. Since we control for six different types of prior publications, we can control for past achievements to explain current productivity net of past productivity, and do so more comprehensively than is typically the case we, and. This also addresses the problems “performance <-> motherhood”, as it measures how motherhood impacts performance before, but, crucially, also after controlling for prior performance (thus measuring the effect of motherhood before and after accounting for selection into motherhood to the degree that it is related to performance). It also takes into account the mentioned reciprocal causality of “earlier performance -> mobility (or grants) -> later performance” as we a) measure the effect of mobility / grants on later performance while *not* adjusting for controls, but then measure the very same relationship while adjusting for prior performance. In addition, we use stepwise regressions that follow our theoretical model in introducing first what cannot conceivably come later, e.g. publications can be related to gender, but gender cannot result from publications. We now mention all of this in the paper.

Note however, that we are limited by the same problems as any study that uses observational data; we cannot be sure that we have accounted for all confounders; in fact, we can be reasonably certain that we could not account for all possible confounders. For example, it is clear that e.g. career commitment is not directly measurable, as it is a latent construct. Not having all variables at our disposal that could potentially explain career advancement is a problem that we share with virtually all studies that rely on observational, and even with many studies that use experimental or quasi-experimental data however. Yet observational studies can still make descriptive (rather than clearly causal) claims. We now mention this as a limitation in the concluding section, asking for intervention studies to confirm or disconfirm our results.

**Reviewer #1**: Second, the text does refer to appendices A-F, but in the document I downloaded are no appendices D, E, or F.
**Author response**: Yes, sorry for this, we have made sure that all appendices mentioned in the paper are also in the appendix.

**Reviewer #1**: Third, the information about the data is incomplete. Just as one example, how has mobility and staying abroad been measured? What data were available got that, and how reliable are these? Please describe into more detail how the variables are measured and whether that is reliable.

**Author response**: We measure mobility as having a received a doctorate from abroad and from the number of months a researcher has stayed abroad; we obtained this information from the researcher’s CVs. In the paper, we have clarified: “We measure international experience through a doctoral degree from an institution obtained outside Germany and through the number of months spent abroad, assuming five months for a semester and ten months for an academic year in case websites did not list exact months.” If specific additional information is required, we would be happy to add it.

**Reviewer comment**: Some other issues: The authors only distinguish three career steps: predoc, postdoc and professor. The last category (and the second too), however, is very heterogeneous and includes everyone from junior/assistant professors to full professors. I would assume that distinguishing junior profs from full profs may have an effect on the outcomes. At least there should have been a convincing argument why this distinction is unimportant.

**Author response**: We agree that splitting up the categories into more fine-grained once is a good idea. We have therefore split up our career stage variable from 3 to 4 categories: 1) pre-doc (no PhD yet), 2) post-doc (PhD, but no habilitation or assistant professorship), 3) assistant professor (habilitation or assistant professorship, but no tenured professorship), 4) tenured professorship (W2/W3). Further splitting up the category of tenured professorship into W2 vs W3 made little sense however, because differences between these two types of tenured professorships are typically small (W2 professors can be better-paid and have more staff than W3) and because professors do not usually tell which of these two types of professorships they have. This is now mentioned in the text.

**Reviewer comment**: The sample includes everyone that got a PhD in psychology since 1980, and that implies a huge variation in age, and even more importantly, in the period they started the career. I would assume that career patterns have changed over time, and that leads to the question how such contextual factors influence the findings. Or the other way around, how is controlled for contextual changes that may have influenced the career patterns?

**Author response**: This is an important point. To take it into account, we have introduced a „cohort“ variable, which accounts for the period during which researchers started their career (measured by when researchers had their first publication).

**Reviewer comment**: Is only the number of publications relevant, or should some other output indicator be preferred, e.g., the number of top cited papers? That would probably be a better indicator, as it includes also the impact of the produced output. And is the analysis sensitive for the selected dependent variable?

**Author response**: We agree that we should have checked this. We therefore added a variable that multiplies each publication by the impact factor of the journal in which it appeared. Results remain essentially the same when doing so.

**Reviewer comment**: Typos (some): (p5) It is therefore reasonable to first test whether more [lacking word?] psychologists acquire more funding in the first place ...
- (p11) nale psychologist = male psychologists?

**Author response**: This has been changed, thank you.

**Reviewer comment**: The article ‘Human capital, research funding, and gender: Determinants of research productivity in German psychology’ aims to analyze the research productivity of psychologists affiliated with German universities. It focuses on determinants of productivity, including previous success in publishing journal articles, gender, institutional environment, international mobility, and grant funding. The study is based on extensive empirical data, which included coding the CV information of all academic psychologists affiliated with relevant departments in more than 70 German universities. The results of the study confirm the cumulative advantage effect suggesting that previous productivity is the most significant predictor of future productivity. The article demonstrates a thoughtful research design, with longitudinal data providing a substantial advantage for causal inferences, and the statistical analysis has been performed appropriately and rigorously. However, several comments may help the authors improve the article. Firstly, the article requires more conceptual clarity. It is unclear whether the focus is on one main determinant or on multiple equivalent factors. Initially, the authors seem to consider two approaches: the sacred spark hypothesis and the cumulative advantage hypothesis. Both approaches conceptualize the prior performance effect differently (as I suppose), though this distinction is not clearly discussed in the theoretical section. While institutional environment, research funding, and gender are important factors that can affect productivity, their roles in the article seem auxiliary as they are considered rather as control variables (“In any case, important intervening variables in any relationship between earlier and later success are a researcher’s institutional environment, research funding and gender, which is why these influences need to be accounted for in any comprehensive model of research productivity”, p.4). This reconstruction is apparent on pages 3-4. However, later in the article, all factors are considered more equivalently, and the initial focus on the clash of two approaches is lost. For example, significant attention is given to gender as a variable. My recommendation is to clarify the role of these factors in the study. It might be beneficial to consider all factors together without focusing on two approaches. If the focus remains on two approaches, it is necessary to clearly define the expected empirical patterns for each approach—specifically, the relationship of the dependent variable with previous productivity or other predictors. Formulating clear hypotheses derived from the two approaches may also bring more conceptual clarity.

**Author response**: Yes, we agree. The structure thus far was not entirely clear. In the introduction, gender was only discussed as one of many variables that might account for productivity; but, as the reviewer mentions, was given more attention later, with our regressions accounting for gender from the beginning. The reason for this is that our two main mechanisms mentioned at the beginning (sacred spark vs cumulative advantage) are likely “gendered”. That is, women may accumulate fewer researcher experience that leads to a slower accumulation of success, which then accounts for the lower productivity of women later on. This is actually what we show empirically. Thus, we have played the discussion of gender after the human capital section in the beginning, now making clear that human capital accumulation may be gendered. In turn, third party funding and institutional environment are confounders, in the sense that acquiring less third-party funding and visiting less prestigious institutional environments might explain why women accumulate human capital more slowly, so we must test the effect of gender before and after controlling for this. We have now not only done so in the paper, but also justified this accordingly. Thank you for making us aware of this disjoint between our empirical analyses and our theoretical discussion, which we could therefore rectify.

**Reviewer comment**: It is also unclear how the authors selected sources to cite when discussing the role of various determinants of research productivity given that this topic has been widely researched. Some cited sources are relevant for the American system, which differs significantly from the European context. I suggest that it is important to address the context of the national academic science system, within which the allocation of rewards and recognition of research achievements play significant roles. Authors may bring more empirical findings from similar national contexts.

**Author response**: We agree that we could have used more sources here. We have added those sources that we have mentioned above in our response to the editor, to show more comprehensively what explains research output and thus also what open questions we can contribute to.

**Reviewer comment**: Furthermore, the placement of international mobility within the institutional environment section may not be appropriate, as the mechanism of mobility may include the expansion of a network of co-authors rather than the effect of institutional place. In other words, such factor as international mobility is broader than institutional environment.

**Author response**: We should have mentioned in the previous version of the paper that when we coded the data, we observed that virtually all international mobility was in fact mobility towards other (international, from a German standpoint) *institutions*. Thus, while one could certainly argue that one important function of international mobility is network expansion, this functions virtually without exception through the international institutions that one visits. This is why international mobility is under the institutional section. We have now mentioned the reasoning behind this explicitly.

**Reviewer comment**: I suggest bring more attention to the discussion of the choice of the main dependent variable. It is worth to put in the main text information from the footnote devoted to the discussion of the two steps: fractionalizing and weighting a publication by journal impact factor (additionally, it seems that the indicated appendix was not placed at the end of the article). There is a consensus in the literature about the hierarchy of publication prestige—papers published in journals of different levels should be regarded as different contribution (see, for example, the discussion in Kwiek, 2023). Given that consensus, it is better to discuss weighting in the main section.

**Author response**: We have moved the discussion of weighting and fractionalizing from the footnote to the main text and inserted the missing tables.

**Reviewer comment**: Justifying the choice of SSCI/SCIE articles in the context of selecting the publication type and the Web of Science is also essential. Given the bias towards English-language publications, it is useful to clarify how common it is for German psychologists to publish in international journals. Is it possible to make a career with virtually no such publications? It would help to understand what part of empirical phenomenon authors are studying.

**Author response**: Indeed, we should have justified more comprehensively why SSCI/SCIE articles are most important. First, we therefore now cite studies that show that SSCI/SCIE articles are by far the most important explanation of getting a psychology professorship in Germany. Second, as a robustness test, we have multiplied all journal articles with the impact factor of the journal in which they appeared, with essentially similar results.

**Reviewer comment**: Although time dynamics are accounted for by design, considering the authors' age—both biological and academic—might be worthwhile, as productivity differences may be associated with cohort and specifics of the academic market over different periods.

**Author response**: While we do not have biological age as a variable (and cannot unambiguously deduce it from official websites), we can (and do) control for “academic age” in the form of career steps, which is highly correlated to biological age (Mayer & Rathmann, 2018, p. 1666). It is a great idea however to additionally control for cohorts, which should also account for specifics of the academic market over different periods. We therefore now control for cohort (defined by having one’s first publication and thus starting an academic career 1) before 1990, 2) 1990-1999, 3) 2000-2009 and 4) after 2009.

**Reviewer comment**: Although, the article indicates similar or different empirical results in various places, wthen discussing results, it would be beneficial to use a paragraph to place the results in the context of similar studies and discuss any similarities or differences. It is also would be useful to provide any suggestions for revealed differences with previous studies. In this regard, I suggest to pay attention to a recent article with similar findings: "Once highly productive, forever highly productive? Full professors’ research productivity from a longitudinal perspective" (Kwiek, 2023).

**Author response**: Thank you, Kwiek was very useful! Except it being an excellent article, we are very glad to be made aware of it, as the article asks to do pretty much what we are doing (“It would be interesting to see whether similar mechanisms operate within social sciences“) and our results confirm the results of this article, notably that prior success is highly indicative of future success. We have cited the article accordingly. We also agree that we should have discussed our results by contrasting them more with the broader literature. We have done so as mentioned above.
