# Supplementary material for: Human capital, gender, institutional environment and research funding: Determinants of research productivity in German psychology
Source: PLoS One. 2025 Feb 12;20(2):e0317673. doi: 10.1371/journal.pone.0317673 (PMC11819567; doi:10.1371/journal.pone.0317673)
Supplement: Attachment — Submitted filename: Response to Reviewers.docx [file pone.0317673.s003.docx]

# Response to Reviewers: PONE-D-24-24540: “Human capital, gender, institutional environment and research funding: Determinants of research productivity in German psychology”

We have copied all suggestions below, detailing how we have taken up each.

**Editor comment**: I would like to ask you to clarify two questions raised by Reviewer 1 and also to add a short non-technical introduction to the three-fold Oaxaca-Blinder decomposition in the methods section. These are only minor changes and I will not send the manuscript back to the reviewers. I'll look at it myself.

**Author response**: We have done all of this as mentioned below.

**Reviewer #1**: The comments of the editor and the reviewers have been taken up by the authors, which has improved the paper. However, a few questions remain. First of all, the text (and earlier papers of the authors) suggest that they deploy an event history analysis. If that is correct it should be said in the methods section, if not the reader would want to have more details about the statistical procedures used, Secondly, the relation between the fixed effects and the random effects may need some additional clarification. In my understanding (but may e the authors use the FE and RE in a different way, as the terms are used differently in the literature), the difference is that RE method allows for random slopes, Whereas the FE model assumes the same slope for each of the higher order entities. If the RE model has a good fit, can one then still conclude something from the FE analysis?

**Author response**: These two points are related, in the sense that the reviewer asks us to explain whether we use event history analysis and to explain which others methods we do use. Event history analysis explains why some individuals are at a higher risk than others of experiencing an event. We have therefore indeed used event history analysis in our papers where we explain who becomes a tenured professor. But we do not use event history analysis here, as we do not explain a (one time) event, but rather the accumulation of publications. Rather than explaining what approach we are *not* using, we explain the approach that we are using. We have therefore slightly enlarged the section that starts with “Last, we use fixed-effects regressions (FE)…”, essentially explain that RE regressions compare researchers, by drawing from “between-individual” and “within-individual” effects, while FE regressions hold between-individual effects constant (fixed) to only show “within individual” effects.

**Reviewer #1**: Thirdly, the three-fold Oaxaca-Blinder decomposition should be introduced in the methods section, as I guess many readers may not know what this is. I feel that these methodological clarifications are needed, but I think this are only minor revisions for the author

**Author response**: Sure, essentially the three-fold Oaxaca-Blinder decomposition shows 1) how much more men publish, 2) whether men publish more because they have *more* of whatever explains higher productivity or 3) because whatever explains higher productivity has a *stronger effect* on the publications of men rather than on the publications of women. I have added this explanation to the methods section.
